# Strengthening the policy setting process for global malaria control and elimination

**DOI:** 10.1186/1475-2875-11-28

**Published:** 2012-01-27

**Authors:** Bianca J D'Souza, Robert D Newman

**Affiliations:** 1 Global Malaria Programme, World Health Organization, 20 Avenue Appia, CH-1211 Geneva 27, Switzerland; 2 London School of Hygiene and Tropical Medicine, Keppel Street, London WC1E 7HT, UK

**Keywords:** global, malaria, policy recommendations, WHO

## Abstract

The scale-up of malaria control efforts in recent years, coupled with major investments in malaria research, has produced impressive public health impact in a number of countries and has led to the development of new tools and strategies aimed at further consolidating malaria control goals. As a result, there is a growing need for the malaria policy setting process to rapidly review increasing amounts of evidence.

The World Health Organization Global Malaria Programme, in keeping with its mandate to set evidence-informed policies for malaria control, has convened the Malaria Policy Advisory Committee as a mechanism to increase the timeliness, transparency, independence and relevance of its recommendations to World Health Organization member states in relation to malaria control and elimination.

The Malaria Policy Advisory Committee, composed of 15 world-renowned malaria experts, will meet in full twice a year, with the inaugural meeting scheduled for 31 January to 2 February 2012 in Geneva. Policy recommendations, and the evidence to support them, will be published within two months of every meeting as part of an open access *Malaria Journal *thematic series. This article is a prelude to that series and provides the global malaria community with the background and overview of the Committee and its terms of reference.

## Background

The World Health Organization Global Malaria Programme (WHO-GMP) has four essential roles [1] (i) to set, communicate, and promote the adoption of evidence-based norms, standards, policies, and guidelines; (ii) to keep independent score of global progress; (iii) to develop approaches for capacity building, systems strengthening and surveillance; and (iv) to identify threats to malaria control and elimination, as well as new opportunities for action.

Last year, WHO-GMP embarked on a major review and re-design of its policy setting process in order to be more responsive to the rapidly evolving malaria landscape. As highlighted in the *World Malaria Report 2011 *published last month, the world is witnessing impressive progress in the development and uptake of malaria control tools, resulting in significant reductions of malaria-related morbidity and mortality in many countries [2]. At the same time, there is increasing pressure on the malaria policy setting process to keep pace with the evidence being generated both through research efforts and the massive implementation of malaria control tools. A stronger and more agile policy setting approach is increasingly important and necessary in the face of a projected shortfall in funding and growing resistance of *Plasmodium falciparum *to anti-malarial drugs and of anopheline mosquitoes to insecticides [2-4]. The global malaria community must make the most effective use of the tools it has in order to meet international targets for malaria control set for 2015 [2].

A small group of independent malaria experts was convened in March 2011 in Geneva to review previous and existing malaria policy processes and successful policy-setting models from other WHO departments. They proposed a framework for a new malaria policy committee - strongly modelled on the Strategic Advisory Group of Experts (SAGE), which sets global policy for immunizations - to address the shortcomings of previous policy processes. During April and May 2011, feedback on the draft terms of reference was sought, received, and incorporated from 50 external stakeholders.

Following approval of the terms of reference by the WHO Director General in August, an open call for nominations was held in September 2011. From the 100 applications received, an independent nomination panel with representation from key partner organizations selected 15 members, who were appointed in November 2011 by the WHO Director General [5].

The inaugural meeting of the Malaria Policy Advisory Committee (MPAC) will take place in Geneva from 31 January to 2 February 2012 in open session [6]. The complete terms of reference for the Committee, outlined in this article, are publicly available online for reference [7].

### Aims and functions of the Malaria Policy Advisory Committee

The mandate of MPAC is to provide independent strategic advice and technical input for the development of WHO policy recommendations on all aspects of malaria control and elimination as part of a transparent, responsive and credible policy setting process.

The MPAC advises the WHO Director-General specifically on:

1. appropriate malaria policies and standards based on programmatic experience by WHO member states and malaria control partners as well as reviews of the best available evidence;

2. engagement of WHO-GMP in malaria-related initiatives;

3. major issues and challenges to achieving global malaria goals;

4. the identification of priority research and control activities to address identified challenges.

### Roles and responsibilities of MPAC members

The MPAC's 15 members serve in a personal capacity and represent a broad range of disciplines, expertise, and experience encompassing many aspects of malaria control and elimination. Members of MPAC, including the Chair, have been appointed to serve for an initial term of three years. Each term may only be renewed once, for a period of up to an additional three years.

The MPAC has no executive or regulatory function. Its role is solely normative; it provides advice and recommendations to the WHO Director General, including response to urgent issues as needed. Members of MPAC play a critical role in ensuring the reputation of WHO in providing high quality, well considered, evidence-informed advice and recommendations on malaria control and elimination. A register of members' declaration of interests is maintained by WHO and will be made available on the WHO-GMP website.

### Meetings and operational procedures

The MPAC will meet bi-annually for three days, with dates generally set at least six months in advance. The frequency and duration of meetings will be adjusted as necessary. MPAC recommendations will be taken by consensus. In the exceptional situation that consensus on a particular issue cannot be reached, the Chair shall report the majority and minority view.

Representatives of the Roll Back Malaria Partnership Secretariat, the Global Fund to fight AIDS, Tuberculosis and Malaria Secretariat, the United Nations Children's Fund and the Office of the United Nations Special Envoy for Malaria have been invited to participate as standing observers in MPAC meetings and deliberations. Relevant staff from WHO Headquarters and Regional Offices will attend as members of the Secretariat. In addition, three rotating National Malaria Control Programme managers from around the world will be invited as resource persons to observe and participate in the meeting.

The MPAC meetings are open to other observers, including representatives from WHO regional technical advisory groups, non-governmental organizations, technical agencies, academic institutions, and donor organizations. Additional experts and technical resource persons may also be invited to meetings to contribute to specific agenda items. Observers will not take the floor unless requested to do so by the Chair and will not participate in the formulation of MPAC recommendations.

The MPAC will work with WHO-GMP to develop its priorities of work and meeting agendas, with input from malaria endemic countries. In time, a wider group will be invited to contribute on agenda items in advance of each meeting via open consultation on the WHO-GMP website.

The MPAC will be kept informed by WHO-GMP and partner agencies of progress in the implementation of strategies and the attainment of objectives at both a country and regional level.

### Evidence review mechanism

Time-limited and specific Evidence Review Groups (ERGs) will be established to review and provide evidence-based information and options for recommendations. These options will be discussed by the full MPAC in sessions open to representatives of stakeholders interested in malaria.

Selected current Technical Expert Groups (TEGs), e.g. the TEG on malaria chemotherapy, will continue to function but will fall under the umbrella of MPAC together with the shorter-term ERGs. The MPAC, together with the WHO-GMP Director, will review the need for existing TEGs, and the creation of new ones, on a regular basis.

### A transparent and timely policy setting process

In order to seek broader input and allow for the exchange of information and views, and to ensure transparency and inclusivity, the majority of discussions will occur in open session. However, the actual deliberations and development of recommendations by the MPAC will take place in a closed session in order to protect the integrity and independence of the committee from pressure and undue influence. Transparency is still ensured however as minutes will be made available on the WHO-GMP website following each meeting, together with the approved MPAC recommendations which will be published within two months of every meeting in the *Malaria Journal*. Approved meeting agendas, minutes, and recommendations will be archived and will continue to remain publicly available and easily accessible on the WHO-GMP website.

This article is the first in what will be a thematic series of policy recommendations to be published in the *Malaria Journal *following every MPAC meeting.

### Conditional policy recommendations

In the absence of a robust evidence base, temporary conditional recommendations, clearly identified as such and based on a combination of the best available evidence and expert opinion, may be issued to provide guidance for regions and countries in the interim period. Conditional recommendations will be reviewed regularly at MPAC meetings in case adjustments need to be made based on newly available evidence.

## Discussion

The call to strengthen the policy setting process for malaria control and elimination so that it is more responsive to the rapidly evolving malaria landscape has been heard. In critically reviewing its policy setting process and implementing changes to increase the timeliness and transparency of its policy recommendations, WHO-GMP is highlighting both its willingness to engage with key partners and its commitment to assist WHO member states in meeting their goals for malaria control and elimination.

WHO-GMP has reached this stage of strengthening the policy setting process for malaria control and elimination through the support of the global malaria community. Convening the MPAC is just the first step in making the policy setting process truly timely and transparent.

WHO-GMP and MPAC will need to continue to engage with the global malaria community in order to successfully fulfil their roles and functions. Further strengthening the policy setting process for malaria control and elimination will involve drawing on the strengths of the global malaria community and the tools in their arsenal. This will include requesting expertise and experience for ERGs and TEGs in order to inform malaria policy recommendations.

## Conclusion

The malaria landscape will continue to evolve. However, change, if anticipated and effectively responded to, can bring about positive transformation. WHO-GMP, MPAC, and the global malaria community as a whole, together have an unprecedented window of opportunity to set policies and programmes in place that will enable them to achieve the ambitious global goals that have been set for malaria.

## List of abbreviations

ERGs: Evidence Review Groups; MPAC: Malaria Policy Advisory Committee; SAGE: Strategic Advisory Group of Experts on immunization; TEGs: Technical Expert Groups; WHO-GMP: World Health Organization Global Malaria Programme.

## Competing interests

The authors declare that they have no competing interests. BD provides part-time secretariat support for the Malaria Policy Advisory Committee to the WHO and is also a part-time DrPH candidate and staff member at the London School of Hygiene and Tropical Medicine. RN is the director of the WHO Global Malaria Programme.

## Authors' contributions

BD and RN conceived the idea for the article. BD wrote a first draft of the article which was edited by RN. Both authors read and approved the final manuscript.
